# Quantitative Field Testing Rotylenchulus reniformis DNA from Metagenomic Samples Isolated Directly from Soil

**DOI:** 10.1371/journal.pone.0028954

**Published:** 2011-12-16

**Authors:** Kurt Showmaker, Gary W. Lawrence, Shien Lu, Clarissa Balbalian, Vincent P. Klink

**Affiliations:** 1 Department of Biochemistry, Molecular Biology, Entomology and Plant Pathology, Mississippi State University, Mississippi State, Mississippi, United States of America; 2 Department of Biochemistry, Molecular Biology, Entomology and Plant Pathology, Bost Extension Center, Mississippi State University, Mississippi State, Mississippi, United States of America; 3 Department of Biological Sciences, Mississippi State University, Mississippi State, Mississippi, United States of America; Auburn University, United States of America

## Abstract

A quantitative PCR procedure targeting the β-tubulin gene determined the number of *Rotylenchulus reniformis* Linford & Oliveira 1940 in metagenomic DNA samples isolated from soil. Of note, this outcome was in the presence of other soil-dwelling plant parasitic nematodes including its sister genus *Helicotylenchus* Steiner, 1945. The methodology provides a framework for molecular diagnostics of nematodes from metagenomic DNA isolated directly from soil.

## Introduction

The sedentary plant parasitic nematode genus *Rotylenchulus* Filip'ev, 1936 is composed of 10 species of tropical and subtropical distribution [1]. In the United States, the major species within this genus affecting crop production is *Rotylenchulus reniformis*, commonly known as the reniform nematode. The only other reniform nematode in the U.S. is *R. parvus* (Williams 1960) Sher, 1961. While *R. parvus* is also found abroad in Mauritius, its distribution in the U.S. is California, Arizona and Florida [2]–[4]. *R. parvus* has not been identified in soils of the other states in the southern U.S. including Mississippi [5]. The *R. reniformis* (reniform) nematode was originally described as a new species in Hawaii [6] that has since spread in its distribution throughout the southern U.S. The reniform nematode has been shown to successfully complete its life cycle on at least 314 plant species, spanning both monocots and dicots [1]. *R. reniformis* is a major crop pathogen in the southern U.S. including Mississippi, Alabama, Louisiana, Arkansas and Georgia with less distribution in South Carolina, North Carolina, Texas, Missouri, North Carolina and Virginia [7]. In the U.S. the reniform nematode causes approximately 100 million dollars in losses, annually [8]. A sufficient threshold of reniform nematodes to cause damage on seedling growth is between 1 and 10 nematodes/cm^3^ soil, [0.8 and 8 nematodes per gram soil at 1.25 specific gravity) [9]–[14]. The rapid spread and agronomic damage caused by the reniform nematode make its identification in soil samples of paramount importance for control and management practices.

With the advent of new and quantitatively reliable molecular strategies, it is possible that the time required to diagnose the reniform nematode in soils could be reduced. Isozyme and DNA-based molecular tests (i.e. PCR) have been developed to determine and to confirm diagnoses of many plant parasitic nematodes [15]–[23]. The PCR-based strategy is also useful because it can provide information on the presence of a particular organism in an environmental sample. Quantitative PCR (qPCR) has allowed for the determination of the number of individuals in bacterial, fungal, and virus populations [24]–[27]. In those studies, the samples were taken from the soil, plant tissues, or nematode samples. Currently, little research has been conducted on the potential of qPCR for diagnostic nematology from environmental samples. However, population estimates for species such as, *Meloidogyne incognita* (Kofoid and White 1919) Chitwood, 1949, *Pratylenchus zeae* Graham, 1951, and *Xiphinema elongatum* Schuurmans, Stekhoven & Teunissen, 1938 have been determined with qPCR on native soil samples from trial plots [28]. The availability of nematode genome sequences for *Caenorhabditis elegans* (Maupas, 1900) Dougherty, 1953 [29], *C. briggsae* (Dougherty and Nigon, 1949) Dougherty, 1953 [30], *Meloidogyne incognita* (Kofold and White, 1919) Chitwood 1949 [31] and *Meloidogyne hapla* Chitwood, 1949 [32] and expressed sequence tags (ESTs) [33] provides a bank of DNA sequences for the development of PCR primers for environmental diagnostics.

The objective of this study was to determine, quantitatively, the number of *R. reniformis* in metagenomic samples isolated directly from the soil, focusing in on soils in Mississippi. An advantage for the studies presented here was that other *Rotylenchulus* species do not exist in soils in Mississippi [5] while at the same time other soil-dwelling plant parasitic nematodes including its sister genus *Helicotylenchus* [34] were present, aiding in comparative purposes.

## Results

### PCR primer and probe design

The study was designed to see if it was possible to determine *R. reniformis* quantities from metagenomic DNA isolated directly from soil (**Table 1**). The β-tubulin gene has been shown to be useful in molecular diagnostics [35], [36] even useful in determining the presence of specific ascomycetous fungi in infected leaf material [37]. The use of the β-tubulin gene in quantitative diagnostic analyses demonstrated that it could possibly be a useful target in determining reniform nematode concentrations in soil samples. From Genbank, a total of 1,119 nucleotide sequences for β-tubulin (758 nucleotide and 361 EST), representing 116 different species were found. This allowed for meaningful comparative analyses for primer design. The Rr-β-TUB gene contains regions that are divergent in primary DNA sequence from other nematode species which facilitated the design of qPCR primers. Importantly, the only member of the genus *Rotylenchulus* that exists in Mississippi soils is *R. reniformis* [5], [7], aiding in the development of primers for *R. reniformis* detection. From those Rr-β-TUB gene sequences, qPCR primers and their Taqman® probes were designed (**Table 2**).

**Table 1:** GPS coordinates of the three SF, Cotton and Corn sites.

Sample	Latitude	Longitude
SF-1	33°25′0.13″N	88°47′17.05″W
SF-2	33°24′57.53″N	88°47′20.27″W
SF-3	33°24′52.73″N	88°47′18.05″W
Cotton-1	33°28′19.83″N	88°46′17.89″W
Cotton-2	33°28′22.03″N	88°46′14.22″W
Cotton-3	33°28′20.54″N	88°46′8.10″W
Corn-1	33°28′43.96″N	88°46′51.46″W
Corn-2	33°28′44.08″N	88°46′53.28″W
Corn-3	33°28′42.94″N	88°46′52.27″W

**Table 2:** *R. reniformis* β-tub primers for qPCR assay.

Name	Sequence
Hit.7. (Rr-β-TUB) F	5′-CAAAATGTCCGCCACCTTCGTT-3′
Hit.7. (Rr-β-TUB) R	5′-GTGCCGTCTCCTCAGCCTCGTA-3′
Hit.7. (Rr-β-TUB) probe	5′-ACGAGATGGAATTCACTGAGGCCGAA-3′

### PCR amplification

**Figure 1:**
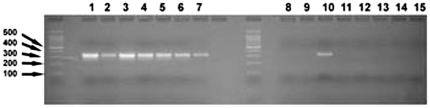
Specificity of the Rr- β-TUB qPCR primers under standard PCR conditions from known numbers of *R. reniformis*. *R. reniformis* DNA was isolated from vermiform J2s serving as the template. The Rr-β-TUB-primed reactions. Abbreviation, R.r. - *R. reniformis*. Lane 1, 1000 R.r.; Lane 2, 1000 R.r.; Lane 3, 1000 R.r.; Lane 4, 100 R.r.; Lane 5, 100 R.r.; Lane 6, 100 R.r.; Lane 7, 10 R.r.; Lane 8, 10 R.r.; Lane 9, 10 R.r.; Lane 10, 1 R.r.; Lane 11, 1 R.r.; Lane 12, 1 R.r.; Lane 13, 1 R.r.; Lane 14, No DNA; Lane 15, No primers.

**Figure 2:**
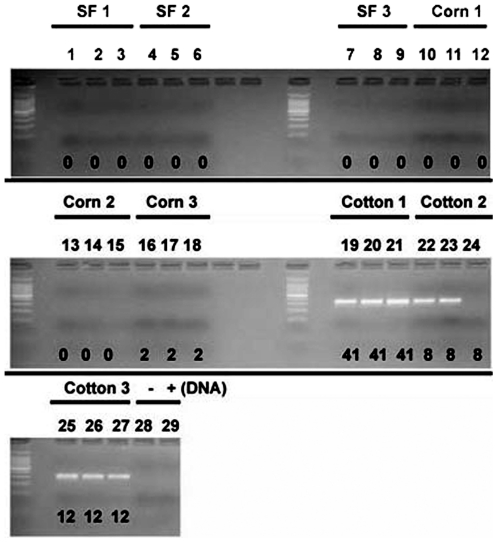
Amplification characteristics of the Rr-β-TUB qPCR primer on DNA isolated from *R. reniformis* extracted from the SF, Corn and Cotton sites under standard PCR conditions. The field sites are SF, Corn and Cotton. Each site was replicated in triplicate. The number of nematodes whose DNA was isolated is provided below the amplicon in each reaction. Lane 1, SF1; Lane 2, SF1; Lane 3, SF1; Lane 4, SF2; Lane 5, SF2; Lane 6, SF2; Lane 7, SF3; Lane 8, SF3; Lane 9, SF3; Lane 10, Corn 1; Lane 11, Corn 1; Lane 12, Corn 1; Lane 13, Corn 2; Lane 14, Corn 2; Lane 15, Corn 2; Lane 16, Corn 3; Lane 17, Corn 3; Lane 18, Corn 3; Lane 19, Cotton 1; Lane 20, Cotton 1; Lane 21, Cotton 1; Lane 22, Cotton 2; Lane 23, Cotton 2; Lane 24, Cotton 2; Lane 25, Cotton 3; Lane 26, Cotton 3; Lane 27, Cotton 3; Lane 28, No DNA; Lane 29, No Primers but having DNA.

**Figure 3:**
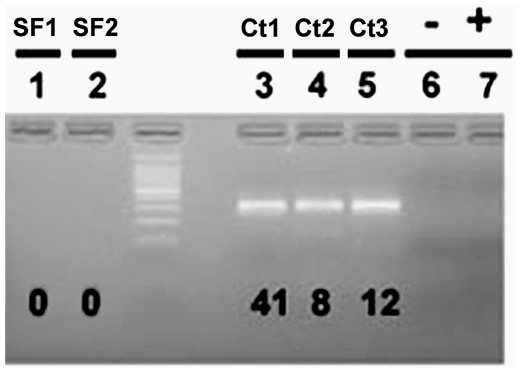
Amplification characteristics of the Rr-β-TUB qPCR primers under standard PCR conditions on metagenomic DNA isolated directly from soil collected at the South Farm (SF) and cotton (Ct) sites. The number of nematodes whose DNA was isolated is provided below the amplicon in each reaction. Rr-β-TUB primed reactions. Lane 1, SF1; Lane 2, SF2; Lane 3, Ct1 sample 1; Lane 4, Ct2 sample 2; Lane 5, Ct3 sample 3; Lane 6, No DNA; Lane 7, No Primers.

The Rr-β-TUB qPCR primers were evaluated in standard PCR reactions with DNA having three different levels of sample complexity. The first level of complexity was pure DNA samples from greenhouse cultured *R. reniformis* (**Figure 1**). The second level of DNA complexity were total populations of nematodes extracted from field soil samples (**Figure 2**). The third and most complex level of DNA were samples from metagenomic DNA isolated from field samples (**Figure 3**). The PCR screen was designed to obtain a quick estimate of the specificity of the primers (**Figures 1** **,** **2** **and** **3**). As expected, when DNA amplified by PCR using the Rr-β-TUB qPCR primers were run out on a gel, the amplification products had a single amplicon and yielded less product with decreasing numbers of starting nematode DNA. Of note, the Rr-β-TUB primers failed to amplify DNA in 2 of 3 samples where a single nematode was used as the template (**Figure 1** **, lanes 8 and 9**), but did amplify in one sample containing DNA isolated from a single individual (**Figure 1** **, lane 10**). The Rr-β-TUB primer never yielded amplification products when no template DNA was provided (**Figure 1** **, lanes 11–13**). The experiments demonstrate that the Rr-β-TUB gene would be a useful gene for detecting nematodes extracted from different field sites to thresholds between 1 and 10 nematodes.

### qPCR of soil extracted *R. reniformis*

Nematodes were extracted from soil samples obtained from the South Farm (SF), Cotton (Ct) and Corn sites (**Table 3**). The DNA from those nematodes was isolated and used for PCR using the Rr-β-TUB qPCR primers. The Rr-β-TUB qPCR primers amplified DNA only in sites where visual identification of *R. reniformis* was made (**Figure 2**). It appears that the threshold for positive identification in soil-extracted nematode samples lies between 2 and 8 nematodes per 500 cm^3^ of soil. Two of the three sites (**Figure 2**, lanes 22 and 23) having 8 *R. reniformis* had amplification (**Figure 2**). However, all three sites (**Figure 2**, lanes 25–27) that had approximately 12 *R. reniformis* yielded amplification (**Figure 2**). All sites with greater than 12 *R. reniformis* amplified *R. reniformis* DNA (**Figure 2**, lanes 19–21) (**Figure 2**). No off target amplification was ever observed under these conditions (**Figure 2**). The specificity of the reaction was determined by sequencing the gel extracted PCR amplified bands, revealing that the amplicon was the Rr-β-tubulin gene (**data not presented**).

**Table 3:** Number of nematodes per 500 cm^3^ of soil used in field assay.

Field Sample Description								
Sample	Crop grown in field	*Rotylenchulus reniformis*	*Meloidogyne* spp.	*Helicotylechus* spp.	*Mesocriconema* spp.	*Pratylenchus* spp.	*Tylenchorhynchus* spp.	Free-Living
1	Fescue	0	0	86	258	0	0	1204
2	Fescue	0	0	86	0	86	0	3698
3	Fescue	0	0	344	0	0	0	0
4	Corn	0	0	86	0	0	0	1634
5	Corn	0	0	0	0	0	86	3526
6	Corn	1743	0	0	0	0	0	1032
7	Cotton	344	0	688	0	0	0	688
8	Cotton	5676	0	0	0	0	0	258
9	Cotton	516	0	0	0	0	0	516

### qPCR of metagenomic DNA isolated directly from soil samples containing *R. reniformis*

The goal of the metagenomic analysis was to take soil samples directly into DNA isolation procedures and downstream qPCR analyses. To achieve this goal; soil samples from the SF sites known to lack *R. reniformis* and Ct sites (always having *R. reniformis*) were focused in on in direct metagenomic DNA isolation procedures. The experiments began by using the Rr-β-TUB qPCR primers under standard PCR conditions as a quick screen to determine how well the primers amplify their target DNA on metagenomic samples. The Rr-β-TUB primed reactions exhibited no amplification in the SF samples (**Figure 3**; lanes 1 and 2). The three Ct sites that had 41, 8 and 12 nematodes, respectively, yielded strong amplification of 300 bp (**Figure 3**; lanes 3–5, respectively).

**Figure 4:**
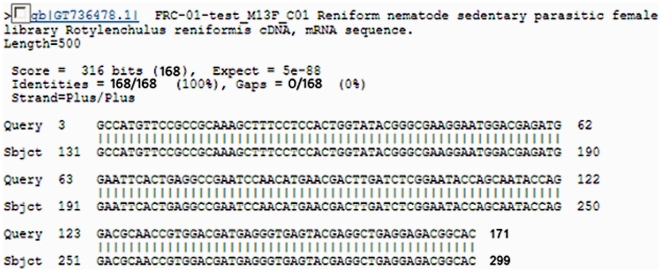
The Rr-β-TUB gene sequence obtained from DNA isolated from a qPCR reaction that was run on an agarose gel, gel extracted, cloned, sequenced and compared to Genbank matching accession GT738331.1.

The obtained specificity of the Rr-β-TUB qPCR primers in standard PCR reactions prompted their use in qPCR reactions with the internal probe. The results show that when using the qPCR primers under qPCR conditions that low concentrations of *R. reniformis* can be quantified (**Table 4**). The results also show that low numbers of *R. reniformis* can be quantified from DNA isolated from field extracted nematodes (**Table 5**) and metagenomic DNA isolated directly from soil samples at three different sites known to either have or lack *R. reniformis* (**Table 5**). The results show a close relationship between the qPCR outcome of hand-counted and *R. reniformis* numbers from DNA extracted directly from soil (**Table 5**). To confirm that the amplification products from the qPCR conditions was indeed *R. reniformis* DNA, the amplicon was isolated, sequenced and compared to the original DNA sequence from which the Rr-β-TUB qPCR primers were designed. The experiment shows the amplification product found in the qPCR reaction was the Rr-β-TUB (**Figure 4**). Thus, the DNA product obtained from the qPCR reactions was amplifying its correct target sequence. The results also show that the limit of detection of hand collected *R. reniformis* is 1 nematode. Furthermore, the detection limit of *R. reniformis* from metagenomic DNA from that same sample is between 2 and 8 nematodes per 500 cm^3^ of soil.

**Table 4:** The Rr-β-TUB qPCR assay estimates of nematodes from a serial dilution of the nematode suspension.

Sample ID	Number of *R. reniformis*	Ct Value Mean	Ct STD	qPCR mean estimate	qPCR STD
1	0	0	0	0	0
2	0	0	0	0	0
3	0	0	0	0	0
4	1	0	0	0	0
5	1	33.25	3.60	21.24	28.29
6	1	37.00	1.03	0.65	0.49
7	10	34.67	0.98	3.00	1.89
8	10	33.19	0.16	7.47	0.83
9	100	31.82	0.49	19.94	6.82
10	100	32.51	1.80	20.40	25.18
11	1000	25.28	0.07	1727.00	82.18
12	1000	25.26	0.20	1754.89	253.59

**Table 5:** Comparison of hand counted (estimated) and qPCR experimentally determined *R. reniformis* DNA extracted directly from soil.

Powersoil® DNA Isolation Sample ID	Estimated number of *R. reniformis*	STD of number of *R. reniformis*	qPCR mean determined *R. reniformis*	qPCR STD
**SF-1**	0	0	0	0
**SF-2**	0	0	0	0
**SF-3**	0	0	0	0
**Corn-1**	0	0	0	0
**Corn-2**	0	0	0	0
**Corn-3**	2.72	0.67	0	0
**Cot-1**	48.63	10.59	16.63	3.47
**Cot-2**	9.73	0.67	8.66	4.00
**Cot-3**	14.00	6.50	13.93	0.69

## Discussion

In the U.S., *R. reniformis* is a major crop pathogen of *Gossypium hirsutum* L. (cotton), *Glycine max* L. Merr. (soybean) and *Ipomoea batatas* L. (sweet potatoes) [7], [38], causing 100 million dollars in agronomic losses, annually [8]. Molecular diagnostic tests have not been developed to determine *R. reniformis* thresholds in soil in the U.S. The current analysis was undertaken to develop a methodology to determine the presence of *R. reniformis* in fields under agricultural practice. The methodology, more broadly, can be used for any biological sample in any environment. The lack of availability of other species of reniform nematode DNA prevented the use of the Rr-β-TUB primers to discriminate *R. reniformis* from the other nine reniform nematode species. However, the soil fauna in the southern U.S. lacks the other *Rotylenchulus* species. It became apparent that this fact [5], [7] may have aided in the development of *R. reniformis* qPCR strategy presented here for most soils in the U.S., including Mississippi. The analysis may have also benefitted by the presence of its sister genus *Helicotylenchus* [34] in the studied soil samples because it served as the closest available relative for comparative off-target analyses.

### β-tubulin as a molecular probe for *R. reniformis* diagnostics

The β-TUB gene has been shown to be useful in molecular diagnostics [35]–[37], providing confidence that it would be useful in formulating a molecular diagnostic for reniform nematodes in Mississippi soil samples. For comparative purposes, a total of 1,119 nucleotide sequences for β-tubulin, representing 116 different species were found in Genbank. Clustalw alignments of the sequence showed areas in which potential primer pairs and probes could be selected. Unfavorable areas that showed high homology, low binding temperature, and repetitive stretches of single nucleotides were avoided in designing primers. The specificity of the primers for Rr-β-TUB was demonstrated for *R. reniformis* in samples where DNA was extracted from as few as 1 individual *R. reniformis*. From this work, it appeared as though the Rr-β-TUB primer pairs could robustly detect the presence of *R. reniformis* in samples where low concentrations of their DNA were present. However, the primer pairs could not repeatedly detect the presence of DNA extracted from a single nematode in all cases. The problem appears to be the ability to efficiently isolate the DNA and not a result of deficiencies in the PCR reaction, itself.

### PCR-based demonstration of metagenomic DNA isolated directly from field extracted nematodes

The goal of the qPCR analysis was to determine, quantitatively, the number of *R. reniformis* in a given soil sample through metagenomic DNA sampling. Since the known threshold of reniform nematodes that are sufficient to cause damage on seedling growth is between 1 and 10 nematodes/cm^3^ soil [9]–[14], the test would have to be at least this sensitive. The soil sites used in the metagenomic sampling either lacked or had *R. reniformis*. In some soil samples, all nematode species were extracted and *R. reniformis* populations quantified, providing a known number of nematodes in the soil samples. The Rr- β-TUB primers demonstrated the ability to amplify a product from soil extracted nematodes that contained between 2 and 8 nematodes. The Rr-β-TUB qPCR assay did not detect fluorescence in samples where no visual observation of *R. reniformis* was made. This observation provided information on using qPCR as a diagnostic tool for *R. reniformis* because it demonstrated that false positives would be of low frequency using this molecular diagnostic analysis procedure. Furthermore, soil from the same samples as used previously to extract all nematode species was also used to isolate metagenomic DNA directly from the soil. In this analysis, the Rr-β-TUB primer pair amplified DNA from metagenomic samples having 8, 12 and 41 *R. reniformis*, respectively. Amplicons from qPCR conditions that were of the expected size were gel-isolated, cloned and sequenced. This precautionary step was done to demonstrate that the amplicons observed from the qPCR conditions were the actual target for the Rr-β -TUB primer pairs. The correct Rr-β-TUB target was confirmed by DNA sequencing gel-purified amplicons from a qPCR analysis on DNA isolated from known numbers of nematodes. The same procedure was used on DNA isolated from nematodes extracted from field samples. The procedure was then repeated on metagenomic DNA isolated directly from soil samples where the number of nematodes had already been counted by visual inspection. Importantly, the metagenomic samples contained its sister genus *Helicotylenchus* (*H. dihystra*) and many other nematodes including *Mesocriconema spp.*, *Pratylenchus spp.*, *Trichotylenchus* spp. and numerous free living nematode species. It is concluded that the primers are specific because DNA amplification was never observed in samples lacking *R. reniformis*, but having these sister species. This work confirmed that the same procedures used to quantify fungal concentrations from leaf samples [37] are applicable to metagenomic soil samples. The data indicate that the use of β-tubulin target gene for determining nematode thresholds from metagenomic samples is a viable option for molecular diagnostics. This is not an unusual application for β-tubulin in molecular diagnostics since the β-tubulin gene has been used in diagnostic tests in other systems [35]–[37]. What is important to note is that a sufficient threshold of reniform nematodes to cause damage on seedling growth is between 1 and 10 nematodes/cm^3^ soil, (0.8 and 8 nematodes per gram soil at 1.25 specific gravity) [9]–[14]. Therefore, the target sensitivity of the diagnostic was to achieve at least this level. The diagnostic studies show that it is possible to reliably detect between 2 and 8 reniform nematodes per 500 cm^3^ of soil sample, an amount that is approximately 55 to 250 times more sensitive than needed. Therefore, the sensitivity of the developed qPCR-based molecular diagnostic using the Rr-β-TUB pair far exceeded the level of threshold of *R. reniformis* nematodes required to cause damage on seedling growth. Nematode levels can also be monitored in near real time during the course of a season.

## Materials and Methods

### Materials statement

No specific permits were required for the described field studies because the soil samples were collected on the North Farm, property of Mississippi State University and contained no endangered or protected species as determined by our soil diagnostic.

### Procurement of cultured, glasshouse-reared nematodes

The target *R. reniformis* and an off-target *M. incognita* were cultured under ambient conditions in a greenhouse at the Mississippi Agriculture and Forestry Experiment Station (MAFES), North Farm, Mississippi State University. Supplemental fluorescent light was provided to bring the day length to a 16 hour day/8 hour night cycle. Temperatures were kept in a constant temperature range between 28.9–34.4°C (84.0–94.0°F). Nematodes were cultured in 500 cm^3^ diameter polystyrene cups (WinCup®; Phoenix, AZ) for a period of 2–6 months in a 50-50 mixture of a Freestone fine sandy loam (46.25% sand, 46.50% silt, and 7.25% clay) and a sandy (93.00% sand, 5.75% silt, and 1.25% clay). Cultures of *R. reniformis*, originally collected from a cotton field located near Tallahatchie, Mississippi (GPS coordinates unavailable), were maintained on the host *Gossypium hirsuta* L. (cotton). Cultures of *M. incognita*, were also originally collected from an infested cotton field near Leflore, Mississippi (GPS coordinates unavailable), and were maintained on *Lycopersicon esculentum* (L.) (tomato). Nematode extractions from plant samples cultured and maintained in the glasshouse were performed according to the procedures of Lawrence et al. [39]. Specifically, vermiform life stages were harvested and separated for each nematode, including *R. reniformis* and *M. incognita*. Soil and roots were submerged in tap water and agitated by hand to loosen the soil from the roots. The nematodes were brought into suspension by hand agitation followed by gravity sieving and sucrose floatation (specific gravity = 1.13). Nematode samples were kept at 4°C until DNA isolations were conducted.

#### Field-extracted nematode sampling

Soil samples from fields in crop production containing many different species of nematodes, including the sister genus *Helicotylenchus* [34] as well as *Mesocriconema* Andrássy, 1965, *Pratylenchus* Filip'ev, 1936, *Trichotylenchus* Whitehead, 1960, *Meloidogyne* Göldi, 1892 and free living nematodes were collected in Mississippi. The targeted collecting was done to test field-isolates of soils that either have or do not have *R. reniformis* which would confirm the sensitivity of the PCR-based studies. Three sampling sites were selected in Oktibbeha County, MS for sampling nematodes for the qPCR analysis (**Table 1**). The sampling sites included (1) a mixed warm season grass pasture where *R. reniformis* has not been found in previous soil sampling, (2) a cotton field known to be infested with *R. reniformis* as determined by previous soil sampling for nematodes from the site and (3) a corn field known to contain low levels of *R. reniformis* as determined by previous soil sampling for nematodes. Four sites, determined to not contain *R. reniformis* through extraction and visual inspection, were also sampled. At each site, metagenomic samples were collected in triplicate. Three 500 cm^3^ soil samples were collected from the top 15.24 cm (6 inches) of soil with a soil probe at the 7 sites as described. The soil was homogenized by hand as previously described. A 150 cm^3^ sub sample of soil was extracted by gravity sieving and followed by sucrose flotation as described previously. The extracted plant parasitic nematodes were identified morphologically utilizing the Inverted TS100 Nikon microscope. Isolated vermiform nematodes were kept at 4°C until the DNA isolations were done.

#### Metagenomic DNA isolation

For metagenomic samples, DNA was isolated from 12 sites taken from 4 fields in which *R. reniformis* was present. An additional 12 sites from 4 fields were sampled in which the nematode was not present as determined by visual inspection and described earlier. Metagenomic DNA isolations were conducted by using the Powersoil® DNA extraction kit® (MO BIO Laboratories, Inc®; Carlsbad, CA). The manufacturer's protocol was followed with modifications. The modifications included the omission of the step that suggested using 0.25 grams of soil in step 1. In its place, 0.3 ml of the nematode suspension, extracted from either glasshouse pots or field soil, was pipetted into the bead beating tube. Secondly, in steps when instructed to remove the supernatant, a standard volume of 400 µl of supernatant was removed from each tube. By doing that step, a standard volume was ensured for downstream applications. The DNA was eluted from the spin column in 100 µl of nuclease free water (Promega®; Madison, WI).

#### *R. reniformis* PCR primer and probe design

The β-tubulin gene has been shown to be useful in molecular diagnostics of organisms in complex samples [35]–[37], providing confidence that it would be useful in formulating a molecular diagnostic for reniform nematodes in Mississippi soil samples. A total of 1,119 β-tubulin nucleotide sequences (758 nucleotide and 361 EST), representing 116 different species were found in Genbank. The comparative analysis database was generated by running format db, with the nucleotide option, from the blastall suite in Genbank (National Center for Biotechnology Information [NCBI] http://www.ncbi.nlm.nih.gov/). The cDNA output from a transcriptome study of a *R. reniformis* adult female that included beta tubulin (Rr-β-tub) (GT736478.1) was downloaded from GenBank [40]. Areas of *R. reniformis* sequences from the ClustalW alignment that had very few matching bases were selected for the *R. reniformis* primer generation. These regions are divergent areas of DNA sequence as described. The sequences were trimmed to the divergent areas and imported into Primerselect® of the Lasergene® software package. Primer pairs were generated using Primerselect®.

#### *Rotylenchulus reniformis* primer pair evaluation

The Rr-β-tub primers (**Table 2**) were evaluated in reactions having three different levels of DNA sample complexity. The first level of complexity was pure DNA samples isolated from greenhouse cultured *R. reniformis* and *M. incognita*, respectively. The second level of DNA complexity was DNA isolated from nematodes extracted from field soil samples. Of note, not all soil samples from the field sites contained *R. reniformis*. The third and most complex level of DNA samples was isolated from metagenomic DNA isolated directly from the field samples. The PCR screen was designed to obtain a quick estimate of the specificity of the primers. For greenhouse and field-extracted nematode samples, known *R. reniformis* DNA was the positive control. The second sample was a negative control of off-target *M. incognita* DNA. As an additional control, metagenomic DNA lacking other *Rotylenchulus* species in Mississippi would also contain DNA from *H. dihystra* (Cobb 1893) Sher, 1961, a species from the sister genus of *Rotylenchulus* [34].

#### Standard PCR reaction conditions

For PCR, a 25 µl PCR reaction consisting of 2 µl template (either *R. reniformis* or *M. incognita*), field extracted nematode DNA or metagenomic DNA, 1.5 µl of 100 nM forward and reverse primers each, 8.5 µl nuclease free water (Ambion) and 12.5 µl Gotaq Green Master Mix (Promega®) was used. The reaction conditions, as reported by Agudelo et al. [41] were modified to include a 2 minute pre-denaturation step at 94°C. The procedure then followed the Agudelo et al. [41] protocol that included a denaturation at 94°C for 45 sec, annealing at 54°C for 45 sec and primer extension at 72°C for 60 sec for 40 cycles. The PCR reaction products were run out by gel electrophoresis on a 1% agarose gel with 0.01% ethidium bromide incorporated into the gel. The DNA amplification products were visualized and recorded with digital imagery using a FOTO/Analyst Apprentice System® (FOTODYNE® Inc.; Hartland, WI).

#### Quantitative PCR reaction conditions

Quantitative PCR (qPCR) Taqman® 6-carboxyfluorescein (6-FAM) probes (MWG Operon; Birmingham, AL) were used. The 6-FAM probes have a maximum excitation at 495 nm and maximum emission at 520 nm. The quencher used in the qPCR reactions was the Black Hole Quencher (BHQ1) (MWG Operon®), with maximum excitation at 534 nm that was used for the analyses. Assays were conducted for primers that produced a single amplicon and had no off target amplification that were determined during the previous screening procedure. The qPCR reaction conditions included a 20 µl Taqman Gene Expression Master Mix (Applied Biosystems®; Foster City, CA), 0.9 µl 100 µM forward primer, 0.9 µl 100 µM reverse primer, 2 µl 2.5 µM 6-FAM (MWG Operon®) probe and 4.4 µl metagenomic template DNA. The conditions were a denaturation at 94°C for 45 sec, annealing at 54°C for 45 sec and primer extension at 72°C for 60 sec for 40 cycles. The qPCR reactions were performed on an ABI 7300 (Applied Biosystems®). Access to the ABI 7300 was kindly provided by Dr. John Brooks, Genetics and Precision Agriculture Research Unit, United States Department of Agriculture-Agricultural Research Service (USDA-ARS), Mississippi State, MS.

To generate a standard curve for the amount of *R. reniformis* in a soil sample, estimates of approximately 1,000 nematodes in 0.3 ml of water were placed into the Powersoil® DNA isolation kit® bead beating tubes and extracted as described previously. A 1∶10 serial dilution series of DNA extracted from approximately 1,000 nematodes was created and used for generation of the standard curve by qPCR. To evaluate the accuracy of the standard curve, samples containing exactly 100, 10, 1, and 0 vermiform *R. reniformis* nematodes were generated by carefully hand collecting them under a stereoscope and isolating the DNA by the Powersoil® DNA isolation kit® as described previously. The qPCR methodology works quantitatively because it detects pathogens by using the amount of DNA present in a sample to obtain a cycle threshold (Ct) value which corresponds to the amount of target DNA [42]. The lower the Ct, the greater the amount of the corresponding DNA (target organism) is present in a sample.

#### Confirmation of PCR and the qPCR amplification products

To confirm that the DNA amplification in both PCR and qPCR reactions were products of *R. reniformis* DNA and not spurious amplification of off-target DNA, DNA amplification products were run out on and then isolated from the 1% agarose gels. The DNA was purified using the Qiaquick Gel Extaction Kit (Qiagen®; Valencia, CA) exactly as according to the manufacturer's specifications. The isolated DNA was ligated into the pGEM®-T Vector System II (Promega®). The ligation reaction was shuttled into competent JM109 cells and selected on 50 µg/ml ampicillin on LB-agar plates. Colonies were selected and grown in liquid culture in LB media containing 50 µg/ml ampicillin. Plasmid DNA was isolated from the bacteria using the Qiaprep kit (Qiagen®). The DNA from the plasmid preps was sequenced to determine if the DNA amplification product was correctly amplifying the proper target. The DNA sequence was trimmed using the Crimson Editing freeware (http://www.crimsoneditor.com/). In this procedure, the pGEM®-T Vector DNA sequence was trimmed leaving the qPCR-generated sequence. The trimmed sequence was blasted in GenBank using the blastn query option. This additional quality control step demonstrated the accuracy of the qPCR reaction conditions.
